# Electronic clinical decision support for children with minor head trauma and intracranial injuries: a sociotechnical analysis

**DOI:** 10.1186/s12911-021-01522-w

**Published:** 2021-05-19

**Authors:** Jacob K. Greenberg, Ayodamola Otun, Azzah Nasraddin, Ross C. Brownson, Nathan Kuppermann, David D. Limbrick, Po-Yin Yen, Randi E. Foraker

**Affiliations:** 1 Departments of Neurological Surgery, Washington University School of Medicine, 660 S. Euclid Ave., Box 8057, St. Louis, MO 63110 USA; 2 Brown School of Social Work, Washington University School of Medicine, St. Louis, MO USA; 3 Institute for Informatics, Washington University School of Medicine, St. Louis, MO USA; 4 Department of Emergency Medicine, University of California Davis, Davis, CA USA

**Keywords:** Sociotechnical analysis, Traumatic brain injury, Head trauma, Electronic clinical decision support, Implementation science, Health information technology

## Abstract

**Background:**

Current management of children with minor head trauma (MHT) and intracranial injuries is not evidence-based and may place some children at risk of harm. Evidence-based electronic clinical decision support (CDS) for management of these children may improve patient safety and decrease resource use. To guide these efforts, we evaluated the sociotechnical environment impacting the implementation of electronic CDS, including workflow and communication, institutional culture, and hardware and software infrastructure, among other factors.

**Methods:**

Between March and May, 2020 semi-structured qualitative focus group interviews were conducted to identify sociotechnical influences on CDS implementation. Physicians from neurosurgery, emergency medicine, critical care, and pediatric general surgery were included, along with information technology specialists. Participants were recruited from nine health centers in the United States. Focus group transcripts were coded and analyzed using thematic analysis. The final themes were then cross-referenced with previously defined sociotechnical dimensions.

**Results:**

We included 28 physicians and four information technology specialists in seven focus groups (median five participants per group). Five physicians were trainees and 10 had administrative leadership positions. Through inductive thematic analysis, we identified five primary themes: (1) clinical impact; (2) stakeholders and users; (3) tool content; (4) clinical practice integration; and (5) post-implementation evaluation measures. Participants generally supported using CDS to determine an appropriate level-of-care for these children. However, some had mixed feelings regarding how the tool could best be used by different specialties (e.g. use by neurosurgeons versus non-neurosurgeons). Feedback from the interviews helped refine the tool content and also highlighted potential technical and workflow barriers to address prior to implementation.

**Conclusions:**

We identified key factors impacting the implementation of electronic CDS for children with MHT and intracranial injuries. These results have informed our implementation strategy and may also serve as a template for future efforts to implement health information technology in a multidisciplinary, emergency setting.

**Supplementary Information:**

The online version contains supplementary material available at 10.1186/s12911-021-01522-w.

## Background

Minor head trauma (MHT) is among the most frequent and most damaging health problems affecting children [1, 2]. While longer-term health effects are one important concern following pediatric MHT, the acute management is largely focused on identifying and managing groups at risk of acute neurological decline [3]. Consequently, there have been several large-scale efforts to develop and validate clinical decision support (CDS) tools guiding the need for computed tomography (CT) imaging in children with MHT [4, 5]. One of these tools developed by the Pediatric Emergency Care Applied Research Network (PECARN) network has subsequently been implemented in several large-scale trials [6, 7].

Head CT decision tools, such as the PECARN rules, offer substantial value to the more than 500,000 children with MHT seen in United States emergency departments each year [1]. However, far less evidence-based guidance is available for managing the 4–14% of children with MHT who are found to have intracranial injuries (ICI) on neuroimaging [5, 8]. While most of these children remain neurologically stable, some will require neurosurgical or other advanced interventions, emphasizing the importance of risk-stratification and close monitoring for high-risk subgroups. At the same time, universal intensive care unit (ICU) admission strains limited resources and may subject patients and families to unnecessary emotional distress [9–11].

Addressing this evidence gap, a provider-facing risk score was recently developed and internally validated using a large-scale multicenter dataset, which is currently undergoing external validation in a large, multicenter cohort [8]. The goal of this risk score is to support evidence-based decision-making (e.g. regarding disposition to an appropriate level-of-care) among pediatric neurotrauma providers at the point-of-care. However, successfully integrating CDS into routine practice remains a challenge, with major barriers including clinician difficulty remembering tool details and absence of a user-friendly interface [13, 14]. Electronic CDS may mitigate many of these barriers by presenting clinician users with relevant evidence at the point-of-care.

Implementing electronic CDS also requires navigating complex sociotechnical dimensions, including areas such as workflow and communication, institutional culture, and hardware and software infrastructure [15]. Failure to investigate these dynamics prior to introducing new health information technology can lead to ineffective efforts and potential unintended consequences [16–18]. Recognizing the importance of such foundational work, we conducted a sociotechnical analysis of electronic CDS intended to promote evidence-based decision-making among clinicians managing children with MHT and ICI. Given our focus on matching patient risk to an appropriate level-of-care, our primary objective was to investigate the sociotechnical influences on implementing electronic CDS to guide clinicians’ decisions regarding the need for ICU admission. Our secondary objective was to investigate alternative uses of electronic CDS among children with MHT and ICI.

## Methods

Leveraging qualitative methods, we conducted a sociotechnical analysis to guide the development and implementation of electronic CDS for children with MHT and ICI. Participants in this study were either physicians who care for children with MHT and ICI, physicians with relevant administrative roles, or information technology specialists experienced in the development and implementation of electronic CDS. All participants were recruited via email solicitation. For the physician participants, we contacted all attending and fellow physicians from neurosurgery, pediatric general surgery, pediatric emergency medicine, and pediatric critical care at a single large academic medical center. Due to the small number of post-residency fellows, we also contacted senior (> post-graduate-year four) residents from neurosurgery and general surgery. To broaden the generalizability of the results, we also solicited participation from attending physicians from these specialties at six other academic hospitals, one community medical center, and one military-affiliated institution (nine total centers). Participants were offered $50 compensation. Interviews were conducted from March through May, 2020.

### Focus group interviews

Individuals who agreed to participate were invited to partake in focus group interviews. Due to the COVID-19 pandemic, all focus groups were held online via Zoom (Zoom Video Communications, San Jose, CA). To avoid imbalances of experience and seniority, physician participants were separated into groups with trainees (residents and fellows) or attendings. We grouped physicians into heterogenous focus groups with representation from different clinical specialties to promote interactive discussions [19]. We also included a dedicated focus group with physicians that also held relevant administrative leadership roles. Non-clinician information technology specialists were organized in a separate group, which included software engineers with focused expertise in CDS graphic design as well as the CDS implementation into the EHR.

**Fig. 1:**
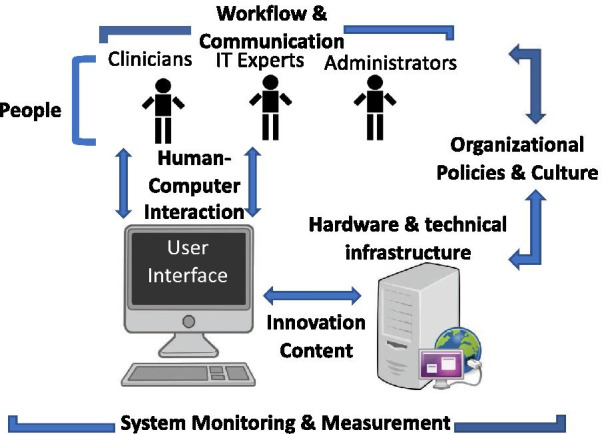
A schematic diagram depicting the domains of sociotechnical analysis [14] investigated during the focus group interviews

**Fig. 2:**
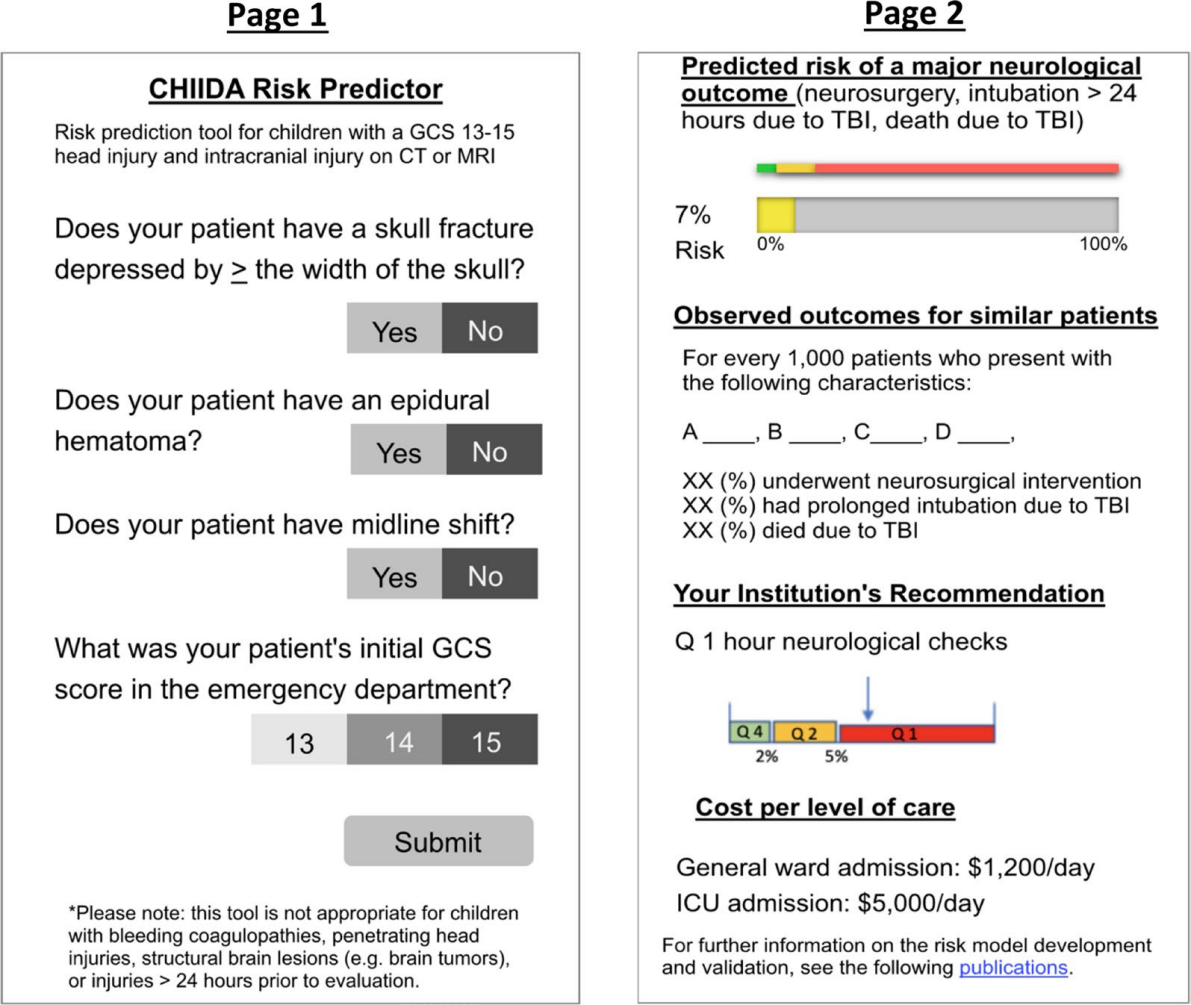
An example of the final wireframe shown to focus group participants

Each focus group was organized in a semi-structured fashion and followed an interview guide developed using input from qualitative methods experts (PYY and RF). The guide (available in the Additional file 1) was intended to investigate key sociotechnical dimensions, including: hardware and computing infrastructure; clinical content; human–computer interface; people; workflow and communication; organizational policies, procedures, and culture; and system measurement and monitoring [15, 20]. These domains are displayed in Fig. 1. Participants were shown a wireframe prototype of the preliminary CDS tool, which displayed predicted risk based on imaging findings and a mental status assessment provided by the Glasgow Coma Scale (GCS) score. While the wireframe was updated across interviews, the final version shown is depicted in Fig. 2. The wireframe depicted an input screen with four questions and an output screen with the following four domains: overall (composite) predicted risk; predicted risk of individual outcomes (e.g. neurosurgical intervention); hypothetical institutional level-of-care recommendations; and costs associated with different level-of-care decisions. A single moderator (JKG, a senior neurosurgery resident with masters-level clinical research training) led all of the interviews, with the aid of a note-taker experienced in qualitative methods (AN and RS). The focus groups were video recorded for subsequent review, and audio recordings were professionally transcribed (Landmark Associated Inc., Phoenix, AZ). Focus groups continued until the research team felt thematic saturation had been reached and no substantially new ideas emerged [19].

### Qualitative analysis

**Fig. 3:**
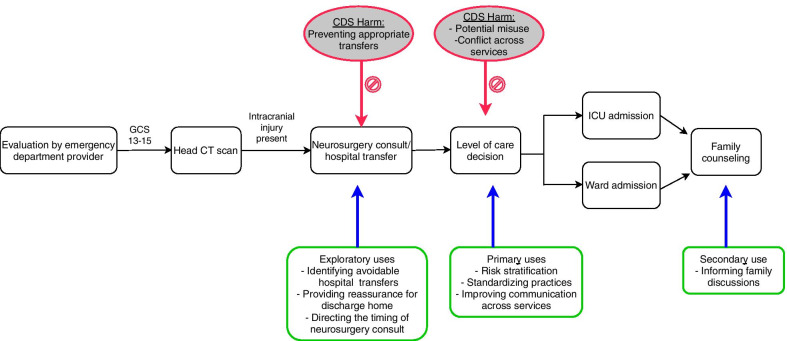
A workflow diagram of the care pathway of children with minor head trauma and intracranial injuries. Points where electronic clinical decision support could facilitate (green boxes) or impede (red ovals) care processes are highlighted

We initially analyzed all focus group transcripts using an inductive process of thematic analysis. [21] First, all focus group transcripts were independently analyzed and coded by two authors (JKG, AO) using Dedoose software version 8.3.35 (Dedoose, Hermosa Beach, CA). A codebook was developed by comparing and reconciling codes and code applications after each transcript in an iterative fashion. These codes were then further modified based on input from qualitative methods experts (PYY and RF). The final coding scheme was applied to each transcript based on consensus agreement between both reviewers, with additional input from other team members (PYY and RF) in ambiguous cases. Next, overarching themes and sub-themes were inductively assigned based on the most important and frequent ideas represented in the interview codes. Based on consensus of both reviewers and qualitative methods experts, final themes were chosen to reflect the most impactful interview results, without seeking to cover every minor comment that was recorded. We then used a deductive process to cross-reference the newly created themes with the defined categories of sociotechnical analysis. Finally, we created a schematic diagram based on the interview results indicating the potential points in the care pathway where electronic CDS could facilitate or impede the management of children with MHT and ICI (Fig. 3).

## Results

We conducted seven focus groups with a total of 32 participants from nine institutions. The duration of the interviews ranged from 39 to 62 min (median 51 min) and included two to six participants per focus group (median five participants). Most participants were either attending (72%) or resident/fellow (16%) physicians, while 13% were non-clinician information technology specialists. Ten (31%) physician participants held relevant administrative positions at their respective institutions (e.g. trauma director, division chief). Most (61%) participants were between 30–39 years and male (59%), and the largest percentage of participants were from either neurosurgery (34%) or emergency medicine (28%). A complete list of participant demographics is shown in Table 1.

**Table 1:** Participant demographics

	Frequency (%)
Age (years)	
30–39	17 (53)
40–49	10 (31)
50–59	4 (13)
Did not answer	1 (3)
Years since completed clinical training^a^	
0–5	14 (50)
6–10	5 (18)
> 10	8 (29)
Did not answer	1 (4)
Gender	
Male	19 (59)
Female	12 (38)
Did not answer	1 (3)
Specialty	
Neurosurgery	11 (34)
Emergency medicine	9 (28)
General surgery	3 (9)
Critical care	3 (9)
Other	2 (6)
Information technology	4 (13)
Training level	
Resident/fellow	5 (16)
Attending	23 (72)
Non-physician	4 (13)
Administrative role	
Yes	10 (31)
No	22 (69)

^a^Non-clinicians were excluded from this calculation. Total percentages do not equal 100 due to rounding of decimals

### Thematic analysis

Across the seven focus groups, 46 unique codes were identified (available in the Additional file 1). These were then inductively grouped into a total of five themes with associated sub-themes: clinical impact; stakeholders and users; tool content; clinical practice integration; and post-implementation evaluation measures. The major themes and sub-themes, along with the corresponding sociotechnical dimensions, are shown in Table 2.

**Table 2:** Major study themes and sub-themes, along with cross-referenced sociotechnical dimensions

Study-based themes	Sociotechnical dimensions
*Clinical impact*	
Helping guide the need for ICU care	Organizational policies and procedures
Informing family discussions	Workflow and communications
	People
Exploratory and controversial uses	Workflow and communications
	People
Unintended clinical consequences	People
	Workflow and communication
*Stakeholders and users*	
Nurses as key stakeholders	People
Use by neurosurgeons versus non-neurosurgeons	People
Importance of multidisciplinary buy-in	People
*Tool content*	
Components	Clinical content
Challenges to using the tool in clinical practice	Not categorized
*Clinical practice integration*	
Integrating the tool within the EHR framework is key but can be logistically challenging	Hardware and software
	Human computer interface
	Workflow and communication
Approaches to targeting users	Workflow and communication
*Post-implementation evaluation measures*	System monitoring and measurement

### Clinical impact

A major topic of discussion related to how the proposed electronic CDS tool could be used in clinical practice. Participants generally supported our primary proposed application of using the tool to help guide the need for ICU care. One participant explained,

*“I think that would have great clinical applications. Just as an example, we looked at our admissions to the PICU [Pediatric ICU] for minor head trauma, and a set of criteria like this would be very helpful, in my opinion, to trying to figure out which patients would benefit from PICU level care...”*

Several participants also explained that the tool would be useful for helping standardize practices and supporting consensus decisions across services. For example, one participant noted,

I also think that anytime where there’s a discrepancy, where you say the patient ought to be in the PICU, and the PICU says ‘no, they ought to be on the floor,’ or something like that, a tool like this may be able to help bridge that gap and help you come to a reasonable decision for the patient.

One neurosurgeon thought the tool would not be useful, expressing,

I’m trying to be open-minded. I’m just not sure how useful it would be. But…I think I’d like to be proven wrong.

Beyond the primary use of guiding the appropriate level-of-care, some participants thought that the tool could also support family counseling. While no physicians endorsed using the tool itself as a shared decision-making aid, several suggested that the risk data provided could inform their discussions with families. One physician stated,

I think it would be good to get the information to us so that we can share it with them appropriately.

Some physicians also endorsed additional “exploratory” uses of the CDS intervention they felt could be appropriate with further validation and acceptance of the underlying evidence. For example, some participants thought the tool could guide the need for hospital transfer or provide reassurance for discharging home some low-risk patients.

Along with these potential uses, participants also identified several unintended consequences that could emerge from implementing the CDS tool. One participant expressed concern that the tool could be misinterpreted or misapplied by non-neurosurgeons.

I’m a little concerned about this being a number that’s going to other services…whenever you give someone a number and say this is your risk, that seems very authoritative if Epic spits that number out to you.

Other participants expressed concern with the notion of using the tool to guide hospital transfers, explaining that community hospitals may be unprepared for a rare deterioration, and may also lack comprehensive trauma services, including care for non-accidental trauma. One physician stated,

if you’re going to use this in outside facilities…it could be used incorrectly if you’re not really careful about it and very explicit in your instructions on how it’s supposed to be used.

Finally, some participants noted that inter-departmental conflicts could also arise if clinicians had differing interpretations regarding the clinical significance of a particular risk score. These potential uses and unintended clinical consequences are summarized in Fig. 3.

### Stakeholders and users

An important area of discussion for physician participants was the tool’s potential use by neurosurgeons and non-neurosurgeons. Several neurosurgeons and non-neurosurgeons supported the idea that the CDS tool would help expand risk knowledge across specialties and training levels, empowering broader groups of providers. For example, one neurosurgeon stated,

I certainly like the best thing about them is being they’re relatively simple…so that anybody from a neurosurgeon to ICU to ER [emergency room] physicians to the pediatricians can evaluate those components to help decide whether you’re going to the ICU or floor or what it might be.

Another emergency department (ED) physician stated,

*“From an ER standpoint, we will still defer to you guys a lot for disposition…but I think just being able to click through one of these would help us figure out what the general risk is.”*

At the same time, several non-neurosurgeons said that the CDS tool had limited relevance for non-neurosurgeons. For example, one ED physician stated,

I’m just not exactly sure what we would do with that information, because if they have a depressed skull fracture, an epidural hematoma, or a midline shift, we’re gonna call…the neurosurgeons anyway.

Despite those differing opinions, multiple participants noted the importance of multidisciplinary buy-in for successful CDS implementation. For example, one physician stated,

I mean I could see it being a problem if we were trying to tell the ER or the trauma services that, ‘hey, look at this algorithm’…and they don’t buy into that.

Outside of the clinical specialties targeted for the focus groups, multiple participants also highlighted the importance of nurses as key stakeholders impacting implementation. For example, some participants noted the need for buy-in from nursing leadership for possible changes in clinical workflow. Additionally, participants felt that nursing capabilities would have an important impact on how the CDS tool is used. For example, one physician stated,

It’s all about how your nurses on the floor or your staff on the floor, what they’re comfortable dealing with and what they’re staffed to deal with.

#### Tool content

Participants provided feedback about the appropriateness of tool components presented in the wireframe, which was then used to refine the initial prototype. There was generally broad support for the predicted risk estimates provided in the tool. One physician explained,

I find the percent risk of going on to need an intervention or be intubated or dying to be the most important thing that you’re putting out here…

Similarly, most physicians were supportive of including general institutional recommendations based on different risk-thresholds, though there was some resistance. For example, one physician said,

the institution recommendation that you have on here. If this says ‘consider ICU admission’ on here, but you and the neurosurgeon talk and you put them on the floor, is there gonna be repercussions if they end up in the ICU?

Another area of concern related to cost estimates associated with different levels-of-care. While some participants saw value in the cost estimates, many others expressed concern with cost information being considered at the point-of-care and also noted that accurate cost estimates (versus charges) could be hard to obtain. One physician explained,

I feel like if the cost considerations have already been assessed by the institution, that information is somewhat enfolded on the institution’s thresholds, in a way, and I wonder if it really needs to be included.

In addition, some participants noted aspects of the tool content that may make it challenging to implement in clinical practice. For example, some participants noted that imaging diagnoses may vary, even among expert radiologists. Additionally, two participants commented that the appropriate timing for assessing the GCS score is unclear (e.g. initial assessment versus worst value), which could impact the values recorded. Other participants noted that some target end-users may lack appropriate expertise to assign all input variables. For example, one ED physician stated,

with regard to the…width of the skull…if I don’t know the answer to that, that would be a very limiting step for me.

Multiple participants also noted that the tool does not evaluate social or non-cranial concerns. For example one physician stated,

I could see a situation where it could may be inappropriately applied in putting someone on the floor that may need a non-neurologic ICU reason [i.e. ICU-level care for non-cranial injuries].

Finally, several participants emphasized that the tool does not capture all of the nuances of clinical decision-making. For example, one physician stated.

I’m sure we’ve all felt that pain of decision support that is evidence-based, and people immediately get hung up on, ‘well the one time this patient had X. Therefore, I need to do this for the rest of the time.’

### Clinical practice integration

All participants generally agreed that to be maximally effective, the electronic CDS tool needed to be integrated within the EHR environment. Participants also emphasized in particular that satellite apps or websites would be a barrier to use, particularly for physicians who may not care for children with MHT on a frequent basis. One resident physician explained,

I think that the optimal place to put it would be in the EHR…If it’s an app that I need to have, or even worse, a website that I need to go to, I’m gonna completely forget about it…

At the same time, several physician participants with informatics experience explained that although building the CDS directly within the EHR is technically feasible, having a website linked to the EHR may be easier to implement. For example, one physician stated,

It’s such an easy thing to do; Epic has the ability and the power to do it, but the logistics of actually pushing it through are so overwhelming that the solution…of doing a web app is actually easier.

While participants generally agreed that the tool needed to be integrated with the EHR, there was less agreement regarding how end-users could best be targeted. Although the IT specialists generally agreed that either a pop-up or a link in the EHR hyperspace were options, participants lacked a clear approach for triggering the tool. Participants discussed linking an alert to an order for a head CT scan or neurosurgery consult, but felt that approach would be non-specific and ineffective. Other participants explained that relying on diagnoses in the EHR would also be challenging, as ED triage diagnoses may not be accurate and ED discharge diagnoses likely would not be recorded sufficiently early in the encounter.

### Post-implementation evaluation measures

The last major theme addressed how participants thought the impact of the CDS should be evaluated after implementation. The complete list of suggested post-implementation evaluation measures is shown in Table 3 and grouped into three categories. First, patient safety/clinical outcome measures included metrics such as unexpected floor-to-ICU transfers that reflected the extent to which the CDS tool improved patient safety. Second, resource use and process measures described changes in the costs following CDS implementation, along with changes in practice patterns (e.g. timing of neurosurgery consults). Finally, participants noted the importance of implementation outcomes, such as how often clinicians actually used the tool when caring for children with MHT and ICI.

**Table 3:** Post-implementation evaluation measures suggested by focus group participants. Measures are grouped in related categories

*Patient safety/clinical outcome measures* Unexpected floor-to-ICU transfers Validating model performance in predicting clinical outcomes (e.g. neurosurgery, intubation) *Resource use and process measures* Number/proportion of ICU admissions Treatment cost Overall length of stay Timing of neurosurgical consults When consults are called Timeliness of consults being seen Inter-hospital transfer rates *Implementation measures* Frequency of the tool being presented and how often it is used Frequency with which the recommendations are being followed	

## Discussion

This report presents the results of a sociotechnical analysis of electronic CDS to aid the management of children with MHT and ICI. Our findings reflect the relatively early stage of this development and implementation effort and also help guide the next steps in this process. The major interview themes covered the anticipated primary and secondary uses of the tool, important stakeholders in implementation, suggestions for improving the tool’s content, along with perspectives on how to integrate the CDS into clinical workflow. Finally, participants highlighted key measures that could evaluate the CDS tool’s impact after implementation.

Despite some concerns and reservations, the focus groups highlighted substantial interest in using the proposed CDS intervention to help guide level-of-care decisions, standardize care, build consensus decisions, and expand risk knowledge across specialties. Although less frequently discussed, there was also support for using the tool to inform family counseling. Participants also raised concerns related to potential unintended consequences to be avoided, such as misuse of the tool to avoid appropriate consults. Another challenge identified related to how risk predictions could be integrated into institution-specific level-of-care recommendations. At present, decision-making varies widely by both physician and institution [8, 22], explaining the need for physician leaders to identify recommendations that reflect institutional culture and capabilities. However, concerns related to blanket recommendations also emphasized the importance of balancing consensus-derived institutional recommendations with physician autonomy in final decision-making, as other similar tools have done [23].

Outside of the primary intended goal of guiding level-of-care recommendations, there was mixed and measured support for other possible uses, such as stratifying the need for hospital transfer. While head injury regionalization is known to vary across the United States, [12] MHT is a major cause of transfer to academic ED’s [24–26], and avoiding some routine transfers may have important implications for distributed trauma systems [9, 27, 28]. Nonetheless, participants explained that changing such practices will require broader acceptance of the underlying evidence. In particular, further prospective validation is needed to evaluate the safety of this application. Likewise, although non-accidental trauma and non-cranial injuries were not identified as important predictors during risk model development [8], these factors must be considered during transfer decisions.

Feedback from the focus groups also impacted the CDS content. For example, the focus groups identified the need to explicitly define the GCS score based on the patient’s first assessment in the treating hospital. Likewise, participant feedback highlighted potential difficulty some physicians may have measuring fracture depression, and a visual aid to assist this task was built into a future prototype of the CDS tool. Finally, the widespread concerns related to including cost information led us to remove that component from future CDS prototypes.

While many of the challenges the interviews identified could be readily addressed, likely the greatest implementation barrier that emerged related to effective approaches of incorporating electronics CDS into clinical workflow. Universally, participants felt that integrating the CDS tool within the EHR was key, and in an ideal setting, CDS elements would auto-populate from the EHR [34, 35], likely improving both clinician satisfaction and CDS use [36]. Likewise, automating CDS alerts within clinical workflow is key to reducing click fatigue and expanding utilization [37–39], which was noted by several participants. While still exploratory, perhaps the most encouraging route to automating CDS use involves the application of machine learning algorithms to detect acute intracranial hemorrhage on CT [44, 45]. Although still requiring physician review, such tools offer a promising avenue for both provider targeting and CDS data capture and should be explored in future efforts.

Beyond identifying new themes, the structure of the focus group interviews was intended to address key sociotechnical elements [15, 20]. We found that the themes and sub-themes identified corresponded to almost all key tenets of sociotechnical theory, suggesting that this model captured most considerations relevant to implementation planning. Given that interface design was not considered in this analysis, the human computer interface dimension was expectedly lacking. In addition, we did not identify any major themes or sub-themes corresponding to external rules or regulations. Likely, this absence reflected the early stage of CDS development, and further evaluation later in the implementation process may yield additional findings. In addition, we found that one sub-theme—“challenges to using the tool in clinical practice”—could not be effectively mapped to a sociotechnical dimension. We believe this difficulty also reflected the early stage of development, where potential problems, such as ambiguous input variables, were still being identified and remediated.

There are limitations regarding our study. First, while we included multidisciplinary physicians, most participants came from academic hospitals, which may limit the generalizability of the results. However, based on the participant feedback, academic clinicians are likely to be the primary targets of future CDS implementation, supporting their higher focus group representation. Second, we did not include nurse practitioners or nurses in the focus groups, who may be potential end-users or stakeholders. Likewise, we did not include lawyers or regulatory experts, whose input could be needed during later stages of implementation to address potential medicolegal concerns raised. Third, given the early stage of the implementation planning, participants reviewed wireframes of the CDS prototype, rather a fully developed prototype. An interactive prototype that integrates the focus group feedback will undergo dedicated user testing in future work. Fourth, more than 80% of our participants were younger than 50 years, and previous efforts have shown older provider age is associated with lower rates of electronic CDS use [46, 47]. Therefore, future larger-scale implementation efforts will need to evaluate potential age-related disparities in CDS use. Finally, we only included physician participants practicing in the United States. Given known differences in EHR use and regulatory structures in other countries, these results might differ if examined in other healthcare systems [48, 49].

## Conclusions

This sociotechnical analysis identified the primary anticipated uses of electronic CDS for children with MHT and ICI, along with other exploratory uses warranting further consideration. By identifying key factors impacting CDS adoption, these results provide a strong foundation for a future implementation trial. This analysis may also inform the development of other electronic CDS tools used in an interdisciplinary emergency treatment setting.

## Supplementary Information

**Additional file 1:** A word document containing the focus group interview guides and a full list of the 46 codes applied to the interview transcripts.

## Data Availability

Due to the confidential nature of the study interviews, copies of the original transcripts are not available. However, researchers with inquiries related to the study methods or results should contact the corresponding author at jacobgreenberg@wustl.edu.

## Abbreviations

GCS: Glasgow Coma Scale
MHT: Minor head trauma
CT: Computed tomography
ICI: Intracranial injury
CDS: Clinical decision support
CHIIDA: Children’s Intracranial Injury Decision Aid
ED: Emergency department
HER: Electronic health record
ICU: Intensive care unit
IT: Information technology
