# Supplementary material for: Electronic clinical decision support for children with minor head trauma and intracranial injuries: a sociotechnical analysis
Source: BMC Med Inform Decis Mak. 2021 May 19;21:161. doi: 10.1186/s12911-021-01522-w (PMC8132484; doi:10.1186/s12911-021-01522-w)
Supplement: Supplementary file 1 — Additional file 1: A word document containing the focus group interview guides and a full list of the 46 codes applied to the interview transcripts. [file 12911_2021_1522_MOESM1_ESM.docx]

**Online-Only Supplement**

**The interview guides used for the focus group interviews.**

Sociotechnical Analysis Focus Group Guide (Clinicians)

**Prior to starting the focus group discussion:**

- Brief study overview
- Individually obtain consent from each participant, as well as contact information for potential follow-up
- Provide individuals an opportunity to ask questions or express concerns in private

**Group overview:**

- Provide an overview of the topic
- Explain the goal of developing an electronic clinical decision tool and the purpose of the sociotechnical analysis
- Explain the audio recording
- Explain confidentiality
- Explain general dynamics for the discussion
- Provide an overview of the sociotechnical model and the different domains that we will evaluate

**Begin focus group discussion**:

1. Ask each participant to introduce him/herself and professional role
2. **Clinical content**
   1. Introduce the CHIIDA score:
   2. What if any role can you see for a tool such as this in the management of these patients?
      1. Potential follow-up: what may make you more or less likely to use this type of tool?
   3. Are there any factors you think are important to add or remove from the tool?
3. **Human computer interface**
   1. Review wireframe of the prototype
   2. Preferred layout of data?
   3. Features that should be added or removed?
4. **Hardware and software computing infrastructure**
   1. Are there any technical factors that you anticipate impacting your ability to use this type of tool?
5. **People**
   1. Which clinicians would find the proposed tool most useful?
   2. Which stakeholder groups should be involved in attempts to implement the proposed tool?
6. **Workflow and communication**
   1. What is the typical clinical workflow for the acute evaluation of children with minor head injury and intracranial injury (*after neuroimaging*)?
      1. Potential follow-up: which clinical services are typically involved?
   2. Where in the care process would the proposed tool be most useful?
      1. Follow-up: which service(s) would benefit most from using the proposed tool?
   3. How could the proposed tool help or impede clinical workflow?
7. **Organizational policies, procedures, and culture**
   1. What if any protocols or practices does your organization currently have that may impact the adoption of the proposed electronic decision support?
8. **System measurement and monitoring**
   1. What are important outcomes to consider when evaluating the impact of the proposed decision support?
9. Conclude focus groups
   1. Thank participants for their time
   2. Ask if there is anything else they would like to add

Sociotechnical Analysis Focus Group Guide (Administrators)

**Prior to starting the focus group discussion:**

- Brief study overview
- Individually obtain consent from each participant
- Provide individuals an opportunity to ask questions or express concerns in private

**Group overview:**

- Explain the study topic, defining the population of interest
- Explain the goal of developing an electronic clinical decision tool and the purpose of the sociotechnical analysis
- Explain the audio recording
- Explain confidentiality
- Explain general dynamics for the discussion

**Begin focus group discussion**:

1. Ask each participant to introduce him/herself and professional role
2. **Clinical content**
   1. Introduce the CHIIDA score:
   2. What if any role can you see for a tool such as this in the management of these patients?
      1. Follow-up: what are potential advantages and disadvantages?
   3. Are there any factors you think are important to add or remove from the tool?
3. **Human computer interface**
   1. Review wireframes of the prototype
   2. Preferred layout of data?
   3. Features that should be added or removed?
4. **Hardware and software computing infrastructure**
   1. Are there any technical factors that you anticipate impacting your ability to use this type of tool?
5. **People**
   1. Which clinicians would find the proposed tool most useful?
   2. Which stakeholder groups should be involved in implementing the proposed tool?
   3. Are there any strategies that you think may support clinician uptake?
6. **Workflow and communication**
   1. What is the typical clinical workflow for the acute evaluation of children with minor head injury and intracranial injury (*after neuroimaging*)?
      1. Potential follow-up: which clinical services are typically involved?
   2. Where in the care process would the proposed tool be most useful?
      1. Follow-up: which service(s) would benefit most from using the proposed tool?
   3. How could the proposed tool help or impede clinical workflow?
7. **Organizational policies, procedures, and culture**
   1. What if any protocols or practices does your organization currently have that may impact the adoption of the proposed electronic decision support?
8. **System measurement and monitoring**
   1. What are important outcomes (clinical or process-related) to consider when evaluating the impact of the proposed decision support?
9. Conclude focus groups
   1. Thank participants for their time
   2. Ask if there is anything else they would like to add

Sociotechnical Analysis Focus Group Guide (IT specialists)

**Prior to starting the focus group discussion:**

- Brief study overview
- Individually obtain consent from each participant
- Provide individuals an opportunity to ask questions or express concerns in private

**Group overview:**

- Explain the study topic, defining the population of interest
- Explain the goal of developing an electronic clinical decision tool and the purpose of the sociotechnical analysis
- Explain the audio recording
- Explain confidentiality
- Explain general dynamics for the discussion

**Begin focus group discussion**:

1. Ask each participant to introduce him/herself and professional role
2. **Clinical content**
   1. Introduce the CHIIDA score and explain how it would be used clinically
3. **Human computer interface**
   1. Review wireframes of the prototype
   2. Are there any design factors that might impact the tool’s integration into the EHR?
4. **Hardware and software computing infrastructure**
   1. What is the process for integrating new tools into the EHR?
      1. Follow-up: which members of your organization are typically involved in this process?
   2. Do you anticipate any technical barriers to integrating the proposed electronic decision support tool?
5. **Organizational policies, procedures, and culture**
   1. How has your organization responded to previous attempts to integrate new decision support into your EHR?
6. Conclude focus groups
   1. Thank participants for their time
   2. Ask if there is anything else they would like to add

**A full list of the 46 codes applied to the interview transcripts**

- A sufficiently validated risk tool could provide reassurance for discharge home
  - Description: A participant statement describing how a tool that is sufficiently sensitive and well validated may be able to identify lower risk patients that could be potentially discharged home.
- CDS content is clinically reasonable
  - Description: Participant statement supporting the clinical appropriateness of the preliminary CDS content.
- CDS supporting consensus among services
  - Description: Participant stated that the CDS tool provides objective data that helps build consensus/create common ground among different clinical services.
- CDS tool expands risk knowledge across specialties and training levels
  - Description: The CDS tool expands knowledge of patient risk to providers who may have less comfort/experience making that assessment.
- CDS tool use depends on skilled imaging evaluation
  - Description: Effectively using the CDS tool requires the provider to be able to independently interpret imaging or have a radiologist's interpretation.
- CDS use in community settings may prevent appropriate transfers
  - Description: Participant expressed concern that implementing the CDS tool in community centers may prevent appropriate transfers to trauma centers.
- Clinical decision making is largely relegated to neurosurgery
  - Description: Statement supporting the notion that non-neurosurgeons largely or entirely defer to neurosurgeons in making management decisions for children with MHT and intracranial injury.
- Design comment
  - Description: Statement reflecting suggestions regarding interface design
- Differing interpretations of CDS output may lead to inter-departmental conflict
  - Description: Differing interpretations of the CDS output by different clinical services could lead to disagreement and confrontation.
- Doubts regarding the clinical utility of CDS
  - Description: Participant statement expressing doubt regarding the clinical utility of the proposed CDS.
- EHR alerts can facilitate use
  - Description: Statement supporting the use of EHR alerts/pop-ups as a means to facilitate CDS use.
- EHR alerts create click fatigue
  - Description: Statement supporting the notion that using an EHR alert to present the CDS would be mentally fatiguing or otherwise poorly received by physicians.
- Expanding focus on evidence-based management
  - Description: Participants describe CDS use as being consistent with broader efforts to adopt evidence-based practices within pediatric trauma/medical management.
- Implementing real-time CDS directly in the EHR is technically possible but practically challenging
  - Description: Participant statement describing the challenges of implementing a tool that runs real-time data analytics (inputs and outputs) directly within the EHR environment.
- Integrating CDS within the EHR will facilitate use
  - Description: Statement describing the need to integrate the CDS tool into the EHR (or a similar electronic format) in order to facilitate clinical use
- Medicolegal concerns may prevent physicians from using CDS guidance
  - Description: Statement describing how medicolegal concerns may prevent physicians from relying on recommendations from the CDS tool.
- Multidisciplinary stakeholder buy-in will promote CDS use
  - Description: Statement describing the importance of getting buy-in from diverse groups of stakeholders to support CDS success.
- Nursing education and buy-in will encourage CDS implementation and
  - Description: Statement describing the importance of educating and obtaining buy-in from nursing to promote successful CDS use.
- Nursing experience and capabilities will change how the CDS tool is applied
  - Description: Statement describing how nursing experience and clinical capabilities will affect the ways the CDS tool is implemented and its guidance is applied.
- Obtaining accurate cost data is challenging
  - Description: Participant statement explaining that obtaining accurate estimates of the costs of care (including who is paying that cost) is complicated and challenging.
- Opposition to including institutional recommendations
  - Description: Participant statement arguing against including institutional recommendations in the CDS tool.
- Organizational procedures for implementing CDS
  - Description: Participant descriptions of individual organizational protocols and processes for implementing new CDS within the EHR.
- Post-implementation evaluation plans
  - Description: Outcomes that need to be recorded/measured/tracked to evaluate the success and unintended effects of implementing the CDS.
- Rare statements/concerns
  - Description: Statements expressed by participants that did not have repeated mention but conveyed potentially important ideas.
- Resistance to changing standard clinical practices
  - Description: Potential resistance to CDS use because of conflict with current establishes practices (e.g. changing standard disposition practices).
- Resistance to including cost data in CDS
  - Description: Participant statement expressing opposition for including cost data in the CDS tool.
- Satellite apps/websites as a barrier to use
  - Description: Any mention of how instituting the tool outside the EHR may negatively impact its use.
- Some CDS input variables may be ambiguous
  - Description: Statements describing potential uncertainty in how clinicians should code some variables in the CDS tool (e.g. ambiguity between SDH vs EDH, assigning a pediatric GCS).
- Suggestions for automating data capture
  - Description: Suggestions on how data needed in the tool can be automatically pulled from within the EHR.
- Suggestions for integrating CDS within the EHR
  - Description: Participant suggestions for how the CDS tool can be presented to users in the EHR.
- Support for including cost estimates in the CDS tool
  - Description: Participant statement supporting the value of including cost estimates in the CDS tool.
- Support for including institutional recommendations
  - Description: Patient statement supporting the inclusion of institutional recommendations in the CDS tool.
- Support for observed risk comparisons
  - Description: Positive opinions about sharing the risk predicted and the actual observed outcomes for similar patients.
- The CDS tool can be implemented with existing technology
  - Description: Participant statement describing how there should not be any significant technological barriers to implementing the CDS tool.
- The CDS tool does not capture all nuances of clinical decision making
  - Description: Statement describing limitations of the evidence underlying the CDS (e.g. how the tool does not reflect every factor that could influence risk).
- The CDS tool does not evaluate social or non-cranial concerns
  - Description: The CDS tool is not designed to incorporate non-cranial injuries or social (e.g. NAT) concerns into risk assessments, but it may still be used to provide a neurological risk score in those populations.
- The CDS tool is a family counseling but not shared decision making aid
  - Description: The CDS tool should not be available directly to families/used for shared decision making. Instead, the output may be used by physicians to inform family counseling.
- The CDS tool is best suited for pediatric trauma centers
  - Description: Participant statement expressing the notion that the CDS tool would be best applied at pediatric trauma centers that have pediatric neurosurgery and other relevant trauma coverage.
- The CDS tool is useful for determining the need for ICU admission
  - Description: Participant statement supporting the use of the CDS tool as a means of deciding the need for ICU admission based on patient risk.
- The CDS tool may be misused by non-neurosurgeons
  - Description: Statement describing concerns related to how non-neurosurgeons may inappropriately apply the output provided by the CDS tool.
- The CDS tool may guide the timing of in-person neurosurgical consults
  - Description: Participant statement reflecting the idea that using the CDS tool may help neurosurgeons evaluate the urgency with which in-person consultations should be seen, in some circumstances.
- The CDS tool provides information primarily relevant to neurosurgeon decision making
  - Description: Statement describing how the CDS tool provides information relevant to the decisions made by neurosurgeons but largely not relevant to the decisions made by other physicians.
- The culture of clinical medicine is conservative
  - Description: Participant statements describing the conservative nature of medicine's culture.
- Typical workflow
  - Description: Examples of typical clinical workflow.
- Using the CDS tool may decrease healthcare costs
  - Description: Participant statement highlighting the potential of the CDS tool to save the hospital or health system money.
- With sufficient validation, the CDS too may guide transfers from community hospitals
  - Description: A participant statement describing how a tool that is sufficiently sensitive and well-validated could help guide transfers from community hospitals.
